# Metabolite profiling of human colon carcinoma – deregulation of TCA cycle and amino acid turnover

**DOI:** 10.1186/1476-4598-7-72

**Published:** 2008-09-18

**Authors:** Carsten Denkert, Jan Budczies, Wilko Weichert, Gert Wohlgemuth, Martin Scholz, Tobias Kind, Silvia Niesporek, Aurelia Noske, Anna Buckendahl, Manfred Dietel, Oliver Fiehn

**Affiliations:** 1 Institute of Pathology, Charité University Hospital, 10117 Berlin, Germany; 2 provitro GmbH, 10117 Berlin, Germany; 3 Genome Center, University of California Davis, Davis, CA, USA

## Abstract

**Background:**

Apart from genetic alterations, development and progression of colorectal cancer has been linked to influences from nutritional intake, hyperalimentation, and cellular metabolic changes that may be the basis for new diagnostic and therapeutic approaches. However, in contrast to genomics and proteomics, comprehensive metabolomic investigations of alterations in malignant tumors have rarely been conducted.

**Results:**

In this study we investigated a set of paired samples of normal colon tissue and colorectal cancer tissue with gas-chromatography time-of-flight mass-spectrometry, which resulted in robust detection of a total of 206 metabolites. Metabolic phenotypes of colon cancer and normal tissues were different at a Bonferroni corrected significance level of p = 0.00170 and p = 0.00005 for the first two components of an unsupervised PCA analysis. Subsequent supervised analysis found 82 metabolites to be significantly different at p < 0.01. Metabolites were connected to abnormalities in metabolic pathways by a new approach that calculates the distance of each pair of metabolites in the KEGG database interaction lattice. Intermediates of the TCA cycle and lipids were found down-regulated in cancer, whereas urea cycle metabolites, purines, pyrimidines and amino acids were generally found at higher levels compared to normal colon mucosa.

**Conclusion:**

This study demonstrates that metabolic profiling facilitates biochemical phenotyping of normal and neoplastic colon tissue at high significance levels and points to GC-TOF-based metabolomics as a new method for molecular pathology investigations.

## Background

Colorectal cancer is the third most common cancer for both sexes, with an estimated number of 153760 new cases and 52180 estimated deaths in the United States in 2007 [1]. Despite advances in diagnosis and surgical treatment of colorectal cancer, incidence as well as mortality of colorectal cancer has decreased only slightly in the last twenty years. While the disease is curable in early stages, the risk of recurrence and metastasis is substantially higher for advanced tumors.

In addition to genetic alterations, the development of colorectal cancer has been linked to a dysregulation of energy homeostasis which is induced by hyperalimentation and adipositas as well as to influences from nutritional intake [2-6]. Recently it has been suggested to use this metabolic deregulation as the basis for new therapeutic interventions [7].

While genetic alterations have been extensively characterized in colon cancer, the metabolic remodeling that occurs downstream from genomic and proteomic alterations have not been analyzed to a great extent, so far. This is in part due to the fact that low molecular metabolites are comparably difficult to analyze in a large-scale -omics approach. Comparably new technologies allow comprehensive and quantitative investigation of a multitude of different metabolites, which is called "metabolomics" in analogy to the terms "transcriptomics" and "proteomics" [8-10] currently, the analysis by gas chromatography (GC) coupled with time-of flight (TOF) mass spectrometry is used as a standard method for studying primary metabolism [11-13]. While these methods are state-of-the-art in the analysis of plant metabolism-, the translation to cancer research for the characterization of tumor types in molecular pathology is still a challenge [14].

For cancer research, the ultimate aim is to define metabolic profiles in normal, precancerous and cancerous tissues and to link the dysregulation of tumor metabolism to clinical outcome and treatment response. To reach this aim, three steps are necessary. First, it is necessary to demonstrate that metabolite levels can be determined in human tissue samples that were collected during routine surgical procedures. Second, it is necessary to identify as many metabolites as possible, to go beyond a simple pattern analysis towards an understanding of the functional alterations of metabolism in tumor tissue. Third, we have to develop tools that allow us to easily interpret metabolic data by connection with the existing knowledge on metabolic pathways that is described in databases such as the Kyoto encyclopedia of genes and genomes (KEGG) database [15].

In human samples, there may be general differences in metabolism that are related to the genetic background or – more likely – to the nutritional intake of the individuals studied. It is therefore not clear, if the metabolic differences between tumor tissue and normal tissue are strong enough to be detected despite the different genetic, metabolic and nutritional background of individual patients.

Therefore, it was one aim of this study to investigate if a set of paired samples of normal colon tissue and colorectal cancer tissue from individual patients can be used for metabolic profiling with GC-MS to detect and interpret molecular changes in tumor tissue and to detect metabolic patterns associated with different biological entities.

We evaluated different bioinformatical strategies to detect those metabolites that are differentially expressed in normal tissue and cancer tissue. To facilitate the interpretation of the results with respect to changes in metabolic pathways, we have developed a new method that projects the metabolite interactions from the multidimensional KEGG interaction lattice to a one-dimensional axis (PROFILE). This method uses the relational information from all metabolic pathways described in the KEGG database but focuses on those metabolites and reactions that can be observed in our investigation and thus builds the bridge to functional interpretation of the metabolomic changes in colon cancer.

## Methods

### Study Population and histopathological examination

For GC-TOF analysis 45 colon samples were examined at the Institute of Pathology, Charité Hospital, Berlin, Germany. The tissue specimens included 27 primary colon carcinomas and 18 normal mucosa samples. For 15 cases paired samples of cancer tissue and normal tissue were available from the same patient. The tissues were dissected by a senior pathologist in the operating room and was immediately frozen in liquid nitrogen and stored at -80°C. Additional H&E sections were performed for histopathological evaluation.

### GC-TOF analysis

Fresh-frozen biopsy tissues (approximately 5 mg fresh weight) were prepared by grinding in 2 ml Eppendorf tubes for 30 s at 25 s^-1 ^using 3 mm i.d. metal balls in a MM300 ball mill (Retsch, Germany). Subsequent extraction was carried using 1 ml of a one phase mixture of chloroform:methanol:water (2:5:2, v/v/v) at -20°C for 5 min. Tubes were centrifuged for 30 s at 14,000 *g *and the supernatant was collected and concentrated to complete dryness. Samples were derivatized for GC-TOF analysis as previously published [11]. In order to avoid cross-contamination of samples, 1.5 μl of the derivatized solution was injected in the split less mode into a thermodesorption unit (DTD, ATAS GL, Zoetermeer, Netherlands) equipped with automatic exchange of liners and micro inserts. The sample was introduced at 40°C using a programmable temperature vaporization OPTIC3 injector (ATAS GL, Zoetermeer, Netherlands) and heated to 290°C using a 4°C/min ramp. Mass spectrometry analysis was carried out using an Agilent 6890 gas chromatography oven (Hewlett-Packard, Atlanta, GA, USA) which was coupled to a Pegasus III time of flight (TOF) mass spectrometer from Leco (St Joseph, MI, USA). A MDN-35 fused silica capillary column of 30 m length, 0.32 mm I.D. and 0.25 μm film thickness was used for separation using a start temperature of 85°C which was ramped by 15°C/min to 360°C. Mass spectra were acquired for a scan range of 83–500 m/z and an acquisition rate of 20 spectra per second. The ionization mode was electron impact at 70 eV. The temperature for the ion source was set to 250°C.

As an additional quality control we used stable isotope labeled cholesterol as internal standard for lipophilic and high boiling compounds for every sample as further control on analytical variation. The subsequent analysis of quality control charts of this deuterated cholesterol standard did not show trends over the duration of data acquisition.

### Data processing and normalization

Two stages of raw data processing were employed. In the initial step, automated peak detection and mass spectral deconvolution was performed by the Leco ChromaTOF software (v2.32). For each sample, around 700 spectra were exported with absolute intensities (peak heights). These spectra were further processed by the in-house programmed database BinBase [16]. All known artifact peaks such as internal standards, column bleed, plasticizers or reagent peaks were excluded from the result sheets. The algorithm underlying BinBase effectively removes inconsistent signals and noise peaks, yielding a total number of 206 metabolic signals that were reliably determined across the entire sample set. Of these peaks, 107 were structurally identified as known metabolites by comparison to mass spectra and retention indices of customized reference mass spectral libraries that were acquired with authentic standard compounds under identical data acquisition parameters. For metabolites that were below detection limits at a given sample, or for which mass spectra did not match the quality criteria underlying the BinBase algorithm, the data set resulted in missing values. In order to minimize the number of such missing values, only compounds were taken into account that were consistently detected in at least 85% of samples. The metabolite data were normalized relative to the sum of the 107 known metabolites in each sample and transformed to the log scale keeping the missing values in the data set. These missing values were further kept in the dataset for metabolite-wise analyses (t-test, fold change calculation). For collective analyses (PCA, clustering, classification) we have replaced missing metabolite measurements by the corresponding arithmetic means over all samples. The entire data analysis was performed within programming and visualization environment R [17]. In additional to the normalization strategy described above, additional analysis were performed using the raw (unnormalized) data as input.

### Principal component analysis (PCA)

Principal component analysis was performed using the function prcomp() in the R package stats. In principal component analysis the original set of metabolites is reduced to a new set of principal components that retain the variance-covariance structure of the data, but use lesser dimensions of data space. Out of the 45 principal components, the fist (PC1) as well as the second (PC2) turned out be significantly different between colon carcinomas and normal mucosa (Welch's t-test, Bonferroni corrected p-values). The values of the first two PCs were plotted with designation of the cases as colon carcinoma or normal mucosa. Further box plots served as visualisation of the discriminative power of the first and the second PC.

### Detection of metabolic changes

Alterations between colon carcinomas and normal mucosa were evaluated by thresholds on the fold change and Welch's t-test p-values. The results of three different selection procedures (p < 0.05, p < 0.01, p < 0.00024) were validated by repeated (n = 1000) random permutations of the samples. The false discovery rates (FDR) for the corresponding metabolite lists were estimated as the ratio (n_exp_/n_obs_) between the number of observed significant metabolites between colon carcinomas and mucosa tissues (n_obs_) and the number of metabolites that were expected to be significant by chance from the permutation distribution (n_exp_).

### Prediction of the tissue type

Predictive models were derived in a two-step procedure consisting of a feature selection step followed by the proper construction of the classifier. We have applied the nearest centroid classification (NCC) and compared it to more complex methods like linear discriminant analysis (LDA) and linear support vector machines (SVM). We used an own implementation of the nearest centroid rule, the implementation of LDA in the R package MAS and the implementation of SVMs in the R package e1071. Feature selection was performed by ranking according to the result of Welch's t-test on the training set. The top 2, 3, ..., 206 metabolites were used as input for the construction of the classifier and classification results were monitored in dependence of the number of features. The classification results were obtained in a leave-one-out approach and reported separately the carcinomas and normal tissues. Beyond that we have studied the stability of the classification results under different choices of the training data. To this end we have employed a protocol similar to that in [28] and trained the classifier on balanced data sets of different size.

### Functional analysis of gene signatures by PROFILE clustering

**Pro**jection **f**rom **i**nteraction **l**attic**e **is a new method for the interpretation of metabolomic changes by the integration of pathway information. Based on the KEGG REACTION data base [cf. ref. [15]] a metabolism network was build by joining two compounds A and B with an edge whenever there was a reaction that converted A into B. Such reaction was assumed to exist if and only if A and B where annotated as reaction pair with the qualifier "main" in KEGG REACTION. The final network consisted of 4206 metabolites that were annotated as "main" in at least one reaction pair. Based on these concepts, the bio-chemical distance between two metabolites was defined as the minimal length of a joining path within the network. The function johnson.all.pairs.sp() from the R package RBGL was used to calculate the bio-chemical distance between all pairs of the 4206 metabolites. The RBGL package is an interface to the Boost C++ library for graph algorithms.

This result was useful for the interpretation of metabolomic changes in colon tissue. Our study included 206 measurements of which 107 could be mapped to chemical compounds and metabolite names. Out of these compounds, 84 were registered in the KEGG database and 71 were a main reaction partner in at least one of the reactions annotated in KEGG REACTION. After projection to these 71 metabolites, the network turned out to be composed out of a single connection component, i.e. pair of metabolites could be joined by a path inside the network. Next we performed an agglomerative hierarchical clustering of these compounds with respect to the bio-chemical distance to project the complexity of the metabolomic network to an one-dimensional axis. In doing so the average linkage method was used to calculate distances between clusters. Finally, the fold changes between cancer and normal tissues were plotted against the functional axis.

## Results

### Metabolic profiling of tissue samples by GC-TOF

**Figure 1:**
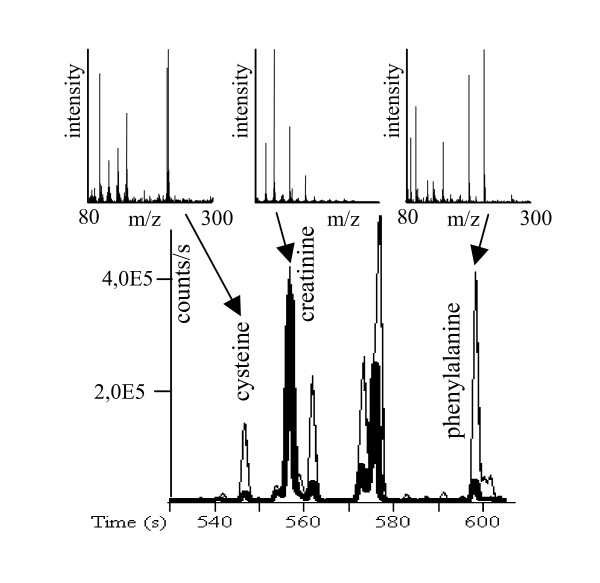
**Representative GC-TOF chromatograms of paired colon carcinoma (thin line) and normal tissue (bold line) at extracted ions m/z 218+100 between 530–610 s.** Select mass spectra (m/z 80–300) are given for the significantly different metabolites cysteine and phenylalanine, and the non-altered compound creatinine.

We investigated a total of 45 samples. 27 of those samples were from primary colon carcinomas, the 18 other samples were from normal colon mucosa. For 15 cases paired samples of cancer tissue and normal tissue were available from the same patient. The clinicopathological data is shown in table 1. With gas chromatography-time of flight mass spectrometry (GC-TOF), around 700 signals were detected per sample using mass spectral deconvolution software for peak detection. However, many of these signals were not consistently found in other samples or were of too low abundance or too poor spectral quality to be unambiguously assigned to unique metabolites. We have hence utilized the novel in-house metabolomic database BinBase that automatically filters out inconsistent signals that cannot be annotate to known metabolites stored in the database using an algorithm exploiting information on retention index, mass spectral similarity and model ions [16]. Moreover, this algorithm automatically adds signals that were not included in the database if detected peaks are consistently found in a relevant subset of samples. All metabolites were then compared against a library of 620 reference spectra of known compounds that were run on the same analytical system, yielding unambiguous identification of metabolites in the database. The complete list of metabolites detected is shown in the supplemental table 1 [see Additional file 1], comprising a total of 206 metabolites that were consistently detected using the BinBase algorithm outlined above under the additional constraint that peaks had to be positively identified in at least 85% of all tissue samples. Metabolites from all major pathways were detected, including TCA cycle intermediates, purine and pyrimidine metabolism, lipids, sugars, amino acids and others. Figure 1 gives examples of the results of the GC-TOF mass spectrometry, demonstrating differences between colon carcinoma and normal tissues.

**Table 1:** Clinicopathological characteristics of the study cohort

Characteristic	Number of cases (%)
**Tissue type**	
Normal colon tissue	18 (40%)
Colon carcinoma	27 (60%)
Cases with paired normal and carcinoma tissue	15 (33%)
**Characteristics of colon carcinomas**	
pT stage	
pT1	1 (4%)
pT2	4 (15%)
pT3	20 (74%)
pT4	2 (7%)
pN stage	
pN0	12 (44%)
pN1	7 (26%)
pN2	8 (30%)
pM	
pMX	26 (96%)
pM1	1 (4%)
DUKES Stage	
A	3 (11%)
B	9 (33%)
C	14 (52%)
D	1 (4%)
Sex	
male	12 (44%)
female	15 (56%)
Histological type	
Adenocarcinoma	30 (100%)

### Unsupervised evaluation of metabolite signatures using principal component analysis (PCA)

**Figure 2:**
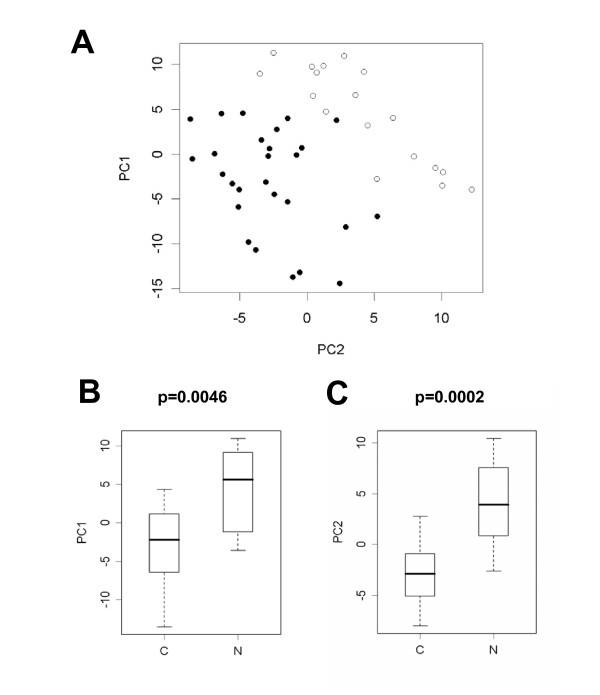
**Principal component analysis (PCA) of colon cancer (dots) and normal colon tissues (circles). **A linear combination of the first and the second principal component leads to a nearly perfect separation of carcinomas from normal tissues (A). A single misassigment is found: one tumor clusters within the normal tissue group. Box plots show the separation of cancer and normal tissues by the first and the second principal component (B, C). P-values shown are from Welch's t-test after Bonferroni correction for multiple testing. The boundaries of boxes and whiskers mark the 5^th^, 25^th^, 50^th^, 75^th^, and 95^th ^percentile.

In a first step, we investigated the global metabolic differences in an unsupervised approach by using principal component analysis (PCA). This approach was chosen because supervised methods tend to over fit models if far more variables than samples are present. In contrary, unsupervised methods do not utilize clinical information about the samples. PCA methods reduce the original set of variables to a new set of principal components that retain the variance-covariance structure of the data, but use lesser dimensions of data space. We analyzed the PCA results with Welch's t-statistics and found the first (PC1) as well as the second (PC2) principle component significantly different between cancer and normal tissue (Bonferroni corrected p-values p = 0.0046 and p = 0.0002, respectively). PC2 yielded a good, but not perfect separation of cancer from normal tissues (figure 2A). However, a combination of PC2 with PC1 allowed a nearly perfect linear separation with a single misassignment, as shown in figure 2B and 2C. A single case of cancer tissue is located in the normal tissue group. These good separation results obtained with unbiased PCA indicate a high potential diagnostic value of metabolic profiles that reflect biochemical differences between normal tissue and cancer tissue.

### Identification of differential metabolites by supervised analysis

**Figure 3:**
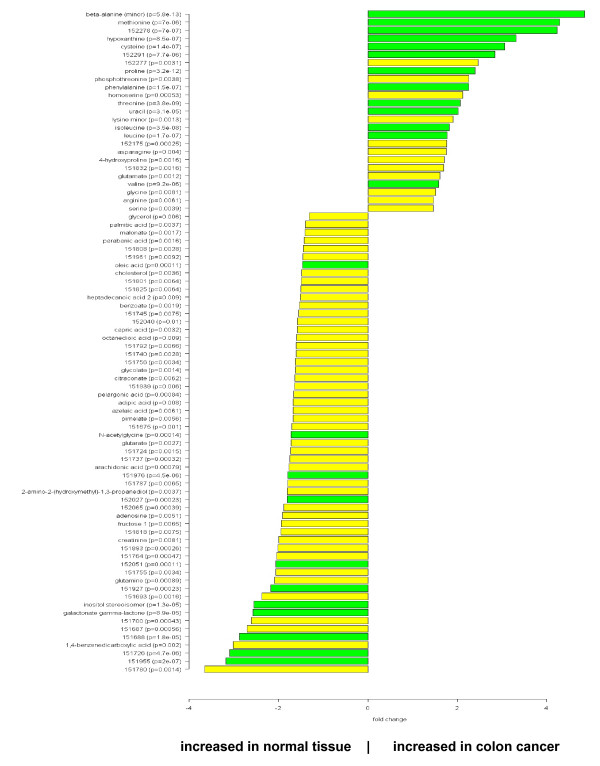
**Comparison of colon cancer versus normal tissue.** Fold change pattern of 82 significantly (p < 0.01) regulated metabolites. p-values are given for each metabolite. Dark columns: metabolites that are highly significantly different even after Bonferroni correction (p < 0.00024).

**Figure 4:**
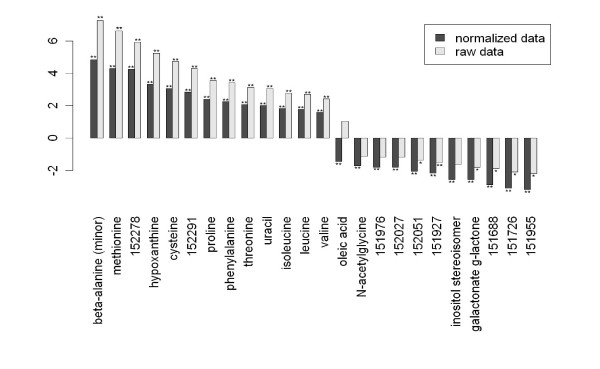
**Evaluation of the influence of normalization.** Raw (unnormalized) data was used for comparison of differential metabolites between normal colon and colon carcinoma. Of the 24 highly significant metabolites 14 (58%) could be confirmed at the high (p < 0.00024, **) and 19 (79%) at the normal (p < 0.05, *) significance level, indicating that the major metabolite differences are not dependent on the normalization strategy.

In the next step we focused on the identification of those metabolites that are differentially regulated between malignant and normal tissue. For this aim, we used a supervised approach based on the Welch t-test to investigate the changes of metabolites in colon cancer tissue in comparison to normal colon mucosa. Using a threshold p-value of p < 0.01, a total of 82 metabolites with significant differences were detected. 25 of these metabolites were up-regulated in cancer tissue, while 57 were down-regulated (figure 3). The complete list of metabolites with fold changes and p-values is given in the supplemental table 2 [see Additional file 1]. The large number of variables measured simultaneously in -omics studies gives rise to a massive multiple testing situation with an accumulating risk of false positive detections. We have addressed these issues in a two-step approach. First, we used the stringent Bonferroni correction. 24 of the metabolites were significantly different even after Bonferroni correction (p < 0.00024, figure 3, dark columns). To investigate the influence of the normalization strategy on the resulting differential metabolites, we have performed an additional analysis using the raw (unnormalized) data as input. Out of the 24 highly significant metabolites 14 (58%) could be confirmed at the high (p < 0.00024) and 19 (79%) at the normal (p < 0.05) significance level (figure 4). These findings suggest that the major metabolite differences are not dependent on the normalization strategy and exclude shifts of the effects between metabolites during data normalization.

**Table 2:** Estimation of false discovery rate and the number of false positives for three different p-value thresholds. The estimates result from a permutation procedure that is detailed in the material and methods section.

p-value threshold	number of detected metabolites	False discovery rate (FDR)	Estimated number of false positive metabolites
0.05	120	8.2%	10
0.01	82	2.3%	2
0.00024	24	0.2%	0

As an additional validation step, we used a permutation analysis to estimate the number of false positives detected by different p-value thresholds. After 1000 permutations of the data set, the number of detected metabolites in all of the shuffled data sets was lower than the number of regulated metabolites between cancer and normal tissues. This was true for all investigated thresholds (p = 0.05, p = 0.01, and p = 0.00024). Finally, the false discovery rate (FDR) and the number of false positive detections were estimated from the permutation analysis, see table 2. Only two false positives are expected within in the list of 82 metabolites that was generated by the threshold p = 0.01.

### Cluster analysis

**Figure 5:**
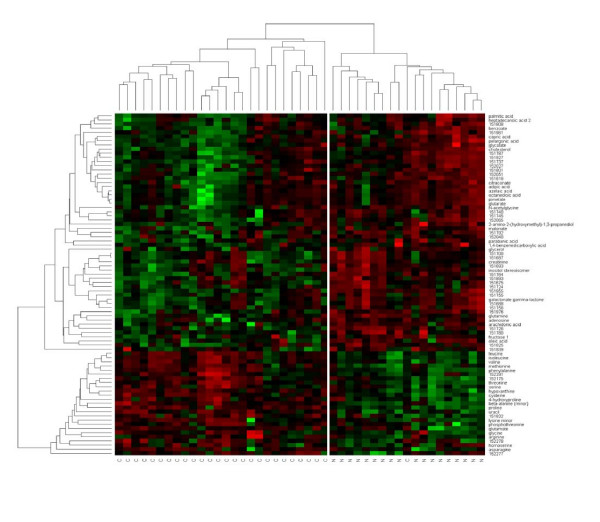
**Supervised clustering of colon cancer tissue and normal colon mucosa with respect to 82 differentially regulated metabolites.** The heatmap visualizes the abundance of each of the metabolites in each of the samples ranging from high (red) over average (black) to low (green).

The results were visualized in a heatmap that was combined with hierarchical clustering of samples and metabolites. Similarity assessment for clustering based on the Pearson correlation coefficient and the average linkage method. Clustering of tumor samples using the 82 significantly regulated metabolites resulted in a nearly perfect separation of cancer tissue and normal tissue, with only one carcinoma clustering in the normal tissue group (figure 5). This case of carcinoma is identical with the single misassignment that has been observed by the unsupervised principal component analysis. Unsupervised clustering using all metabolites did not result in a complete separation between carcinomas and normal tissue (data not shown).

### Classification models for prediction of tissue-type

**Figure 6:**
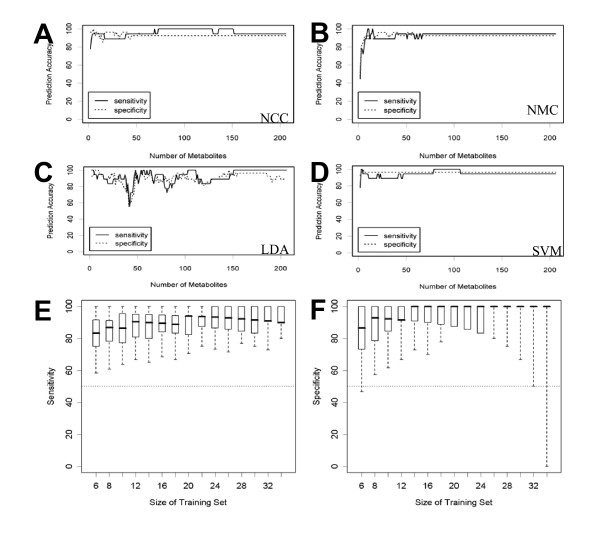
**Validation of predictive models for the separation of colon cancer from normal tissues based on 2, 3, 4, ..., 206 metabolites. **Sensitivity and specificity were calculated in a leave-one-out approach. **A: **nearest centroid classification (NCC), **B: **nearest mean classification (NMC), **C: **linear discriminant analysis (LDA), **D: **linear support vector machines (SVM). **E/F: **Validation of nearest centroid classification (NCC) with 206 metabolites by looking at training data sets with n = 6, 8, ..., 34 samples. For each size n, 1000 training data sets were generated by randomly drawing n/2 tumors and n/2 normal tissues from the 45 samples. The remaining 45-n samples were used as test data. Sensitivity **(E) **and specificity **(F) **were reported and their distributions were visualized as box plots. The boundaries of boxes and whiskers mark the 5^th^, 25^th^, 50^th^, 75^th^, and 95^th ^percentile.

Different supervised methods were employed in order to construct predictive models that are capable of distinguishing between colon carcinoma and normal tissue based on metabolomic profiling [18-20]. We used the nearest centroid rule for classification of normal tissue versus carcinoma. This simple prediction rule classifies a tissue according to its Euclidean distance to the average mucosa and the average carcinoma signature; the predicted category is that with the smallest Euclidean distance. Nearest mean classification (NCC) was conducted for signatures containing 10, 25, 50, 100 and 206 metabolites and validated in a leave-one out approach. As shown in table 3, for each classifier the sensitivity and the specificity for detection of cancer was detected. All classifiers had sensitivity and specificity around 95%. Classification with more complex decision rules as linear discriminant analysis (LDA) or support vector machines (SVM) did not lead to an improvement of classification accuracy (figure 6A–D). However, our results show that metabolic profiling can be used to classify tissues and to distinguish between normal and cancer tissue with high accuracy. Classification results were robust with respect to different choices of the training data set (figure 6E, F).

**Table 3:** Sensitivity and specificity for the detection of colon cancer with the nearest centroid classifier based on the levels of 10, 25, 50, 100 or 206 metabolites.

number of metabolites in the classifier	sensitivity	specificity
10	96%	94%
25	96%	89%
50	93%	94%
100	93%	100%
206	93%	94%

### Interpretation of metabolic changes in tumor tissue using pathway information from the KEGG database (PROFILE)

Metabolic profiling analyses have the advantage that reliable and standardized information on metabolic pathways exists in the KEGG database. However, with the current techniques in data acquisition and deconvolution it is not possible to measure all the metabolites corresponding to each pathway. Many compounds involved in pathways are intermediates with too low concentrations to be reliably detected. Others are involved in more than one pathway, depending on the localization and distribution between the cellular compartments. KEGG can be interpreted as a multidimensional metabolic network that can be visualized by the KEGG pathway diagrams. However, these diagrams are only partially suitable for visualization and interpretation of metabolic results, since the detected metabolites usually come from different pathways and not all metabolites from a single pathway can be detected. Furthermore, the various cross-links between different pathways that may be relevant as well are very difficult to visualize with the conventional approach of using full KEGG pathway maps. Efforts have been carried out to reduce complexity by constructing static pathway maps [21]; however, such approaches are not flexible enough to integrate novel information and are too superficial to depict the different roles and connections of metabolites in metabolic networks. In previous reports [22] we have proposed to exploit differences in metabolic network graphs. On the one hand, such graphs are powerful because both identified and unidentified metabolic signals can be taken into account. On the other hand, the biological relevance and pathway connection of unknown metabolites cannot be known. Therefore, we constrain analyses here to only structurally identified compounds that can be mapped to pathways.

We have developed a new approach to visualize metabolomics data in context of the KEGG database. PROFILE is based on projection of the metabolite interactions from the multidimensional KEGG interaction lattice to a one-dimensional axis. PROFILE starts with the calculation of the metabolic distance of each pair of metabolites in the KEGG interaction lattice. This first step is performed using all metabolites in the KEGG database, without a restriction to those that can be actually measured. Distances between all metabolites are described as the number of main biochemical reactions that are needed to connect these metabolites. The result of this first step is a 4206 × 4206 data matrix that represents metabolite distance across the complete KEGG REACTION database. In a subsequent step, this matrix is reduced to visualize relationships between those metabolites that can actually be measured by GC-TOF profiling of colon tissues.

**Figure 7:**
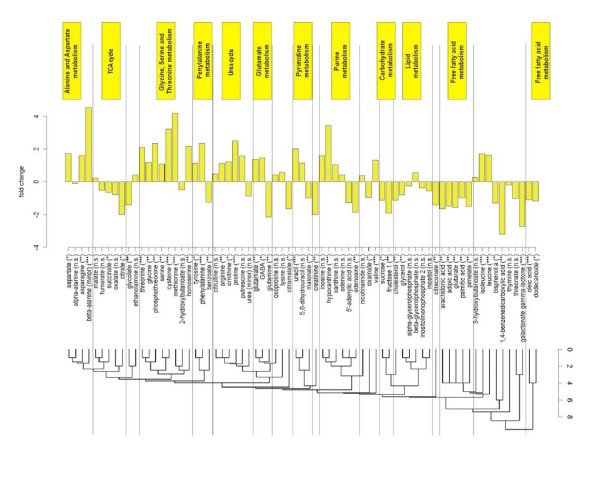
**PROFILE analysis – comparison of metabolic differences between colon cancer and normal tissue clustered according to the relational pathway information from the KEGG REACTION database. **Significance of the metabolic differences is designated by the stars behind the metabolite names: *** (high significance, p < 0.00024), ** (medium significance, p < 0.01), * (low significance, p < 0.05). The scale at the dendrogram refers to the number of main reactions in the KEGG database that link the metabolites to each other.

Our study included 206 metabolic signals of which 107 could be identified by chemical structures and metabolite names. Out of these compounds, 84 were registered in the KEGG database and 71 were a main reaction partner in at least one of the reactions annotated in KEGG REACTION. In the next step these 71 metabolites were clustered with respect to the distances derived from the KEGG REACTION database. The resulting PROFILE clustering is shown in figure 7 with links to the corresponding biochemical pathways and key enzymes in supplemental table 3 [Additional file 1]. As absolute concentrations of different metabolites in human tissues can differ by orders of magnitude, we found it especially useful to plot fold changes between cancer and normal tissues along the functional axis.

As shown in figure 7, the PROFILE method clusters many compounds according to major metabolic pathways such as urea cycle, purines or TCA cycle intermediates. Within these pathways, compounds are usually deregulated in the same direction, i.e. urea cycle intermediates, purines and most amino acids up regulated in carcinoma tissues and TCA cycle intermediates and free fatty acids down regulated. Consequently, the clustering of results according to metabolic distances helps annotating significant differences in regulation to pathways, in contrary to the alternative display of metabolic differences by p-values or by fold-changes. Interestingly, the most significant metabolic difference was found for up regulation of beta-alanine in colon carcinoma.

## Discussion

Our study demonstrates that large scale metabolic profiling using GC-TOF mass spectrometry and database annotation yields numerous significant differences between colon carcinoma and normal colon mucosa. We have utilized both unsupervised and supervised approaches to investigate these metabolic differences. The metabolite signatures are capable of predicting the status (normal tissue or colon carcinoma) of a previously unknown test tumor at sensitivity and specificity around 95%. Importantly, we could show that the classification results are robust against different choices of the classificator and the training set (figure 6). Regarding the extent of changes detected, it is important to note that – from a tumorbiological point of view – the comparison of normal tissue and carcinoma tissue means that two completely different entities are compared. Therefore we would expect that comparison of those two tissue types leads to a large set of completely different biomarkers. Similar results have been reported in gene expression analysis. For example in the study by Hlubek et al. [23] 39% of transcripts were differentially expressed between colon tumor center and colon normal tissue. Since metabolites are regarded as an amplified output of a biological system, the expected metabolite changes could be even more prominent compared to genomics, as shown in our analysis.

Many of these metabolic events can be ascribed to known metabolic dysregulation in cancer thus validating the method itself. Metabolites involved in the citric acid cycle were generally found at lower amounts in cancer tissues compared to normal colon samples, in accordance with results published earlier [24]. Purines were detected at increased levels in malignant tissues as indicator for higher capacity for DNA synthetic capacity. Similarly, we found almost all amino acids to be up regulated in carcinoma tissues, which may be interpreted as reflecting cellular needs for higher turnover of structural proteins. This finding is in agreement with earlier publications for select amino acids, notably glutamate and aspartate [25]. Similarly, the high GABA contents had been described in colon cancer tissues in a previous study [26]. Certain amino acids are synthesized by mammalian metabolic routes, often using TCA intermediates as precursor such as alpha-ketoglutarate for glutamate and its derived amino acids, and oxaloacetate for aspartate-derived amino acids. With higher needs in amino acids but lower use of the TCA cycle, an alternative route is needed to deliver carbon backbones for such TCA-derived intermediates. Such higher import may be accomplished by up regulation of amino acid transporter, facilitating higher cellular needs for energy metabolism as well as delivering carbon backbones for biosynthesis of cellular molecules. This interpretation is supported by our finding of increased levels in urea cycle intermediates in colon carcinoma tissues, indicating higher turnover of amino acids. Interestingly, beta-alanine was found as the most upregulated (f.c. = 4.9) metabolite in carcinoma tissues with very high statistical significance (p = 5.8e-13). In humans, beta-alanine is a unidirectional catabolic product from aspartate in a decarboxylation reaction (EC:4.1.1.15) or by catabolic routes from pyrimidine metabolism (EC:3.5.1.6). However, the eventual fate of beta-alanine in humans is yet unclear, since no enzymes are known that would transfer its backbone into acetyl-CoA or towards pantothenate metabolism, as it occurs in other species. We therefore suggest that beta-alanine might be important for metabolic alterations in colon cancer. In addition, we found that not all amino acids were up regulated in the same manner. In fact, the glutamate/glutamine ratio was greatly altered in comparison to normal colon tissue, indicating a lesser role of aminotransferase reactions utilizing glutamine or less need for transport of nitrogen across cells.

A limitation of this study was found in the need of normalizing the raw data to the total sum of known metabolites. The normalization strategy was developed in analogy to gene expression studies and was chosen because frozen tissue sections (as detailed in the methods section) should not be weighed on fine balances in order to preserve the cold chain and to prevent reactivation of metabolism prior to extraction. We found that the raw data for carcinoma tissues were significantly higher (p = 0.03) than those for normal tissue, relating to roughly a 33% increase in overall metabolic levels. However, this might be due to either a higher number of tumor cells per area of tissue or for generally enhanced metabolism. More detailed studies would be needed to address this question. In an additional validation using the raw (unnormalized) data as input the major metabolic differences between both tissue types could be detected, as well, suggesting that the major metabolite differences are not dependent on the normalization strategy.

The total time between surgery and freezing tissues was kept as minimal as possible due to the fact that the frozen section pathology laboratory was directly adjacent to the operating room. Nevertheless, clinical and pathological workflows do not allow for exact measures and timing of tissue dissection parameters for samples collected during routine surgical interventions. In addition, depending on the surgical technique there is a variable amount of intraoperative tissue ischemia due to surgical ligation of blood vessels. This fact may account for the inability to quantify glycolytic intermediates which have such a high turnover in non-frozen tissues that these are found to be depleted if metabolism is not immediately quenched after disruption of blood flow. To minimize unrelated technical noise related to surgical procedures we have chosen to compare tumor tissue and normal tissue collected during the same surgery.

We have used a metabolomic approach by GC-TOF mass spectrometry in order to gain a broad overview over primary metabolism at limited costs but at high sensitivity and selectivity. In total more than 100 compounds could be identified by chemical structure from as little as 5 mg fresh tissue, which compares favorably to reports using one dimensional ^1^H-NMR data acquisition. Specifically, we here demonstrate for the first time the efficacy of an automated annotation using a customized database approach. On the one hand, the BinBase database unambiguously identifies chemically or biochemically known compounds that are utilized for pathway mapping. On the other hand, the database also facilitates adding novel and potentially unique metabolic signals that yet are to be structurally identified but that nevertheless were often found to be differentially regulated at high significance levels. These compounds are stored in the database by unique identifiers combining mass spectra and retention index information that enable re-using these database entries for later studies aimed at validating initial biomarkers or at structural identification of these metabolic signals.

In the study presented here, we have focused on using information from identified compounds by developing and applying a new biochemical mapping method, PROFILE. This method maximizes the interpretability of results by facilitating physiological and biochemical understanding of metabolic alterations in carcinoma. Specifically, PROFILE leads to simplified output of results than mapping on single pathway maps from KEGG which would focus on a small number of select metabolites rather than taking into account the relative distances of metabolites across the metabolic network.

As a conclusion, our results show that metabolic signatures as well as individual metabolites can be detected from fresh-frozen tumor tissue of colon cancer and that these alterations can be linked to relevant biochemical pathways. Based on our results, we suggest that metabolomics is a promising approach complementary to transcriptomics and proteomics for analyses of changes in the malignant phenotype. As metabolites constitute the amplified output of a biological system, their quantitative and qualitative analysis will be relevant for tumor biology in different types of investigations. Databases such as the one presented here will enable comparisons of findings across studies and laboratories. Metabolomics can be used for biochemical classification of different tumor types and for comparison of malignant tumors with their corresponding normal tissue. Recently, it has been suggested that therapeutic approaches directed against metabolic abnormalities may be useful in the treatment of malignant tumors [27,28]. In this context, the metabolic profiling approach described here may be useful to monitor the complex changes in tumor metabolism that may occur under these treatments. Furthermore, analysis of metabolic alterations may be used as a new method for molecular pathology to develop classifiers for therapy response prediction, which may ultimately lead to the identification of new prognostic markers. With the combination of advanced instrumentation, standardized database algorithms and the development of tools for interpretation of data, our study provides a methodological basis for these further investigations.

## Competing interests

The authors declare that they have no competing interests.

## Authors' contributions

CD conceived the study and participated in its design, participated in the statistical evaluation, performed the histopathological evaluation and helped to draft the manuscript. JB performed the profile clustering and the statistical evaluation and was involved in drafting the manuscript. WW carried out the design of the study and performed the histopathological evaluation. GW, MS, and TK performed GCTOF analysis and the identification of metabolism. SN, AN, and AB helped to draft the manuscript and participated in the histopathological evaluation. MD participated in the design of the study. OF participated in the design of the study, performed statistical analysis and did GCTOF analysis and performed the identification of metabolism. All authors read and approved the final manuscript.
